# Small Bowel Injury During Peritoneal Entry at Cesarean Section: A Five-Year Case Series

**DOI:** 10.7759/cureus.31072

**Published:** 2022-11-03

**Authors:** Husham A Ahmed, Zeena S Bu Shurbak, Isaac A Babarinsa, Huda A Hussain Saleh, Najat Khenyab, Zia Ahmed, Fathima Minisha

**Affiliations:** 1 Obstetrics and Gynecology, Women’s Wellness and Research Center, Doha, QAT; 2 Surgery, Hamad General Hospital, Doha, QAT

**Keywords:** consent content, risk management, cesarean section, peritoneal entry, small bowel injury

## Abstract

Introduction

Small bowel injury during peritoneal entry may occur unexpectedly at cesarean section (CS) and may present unexpected management problems and prolonged postoperative hospital stay.

Methods

This was an observational study of patients who sustained inadvertent injuries compared to those who did not. Both study and control patients had the same number of previous cesarean sections.

Findings

In this study population, the frequency of small bowel injury during peritoneal entry was 0.0003/10,000 cesarean sections. The majority comprised serosal trauma (7/10) and tended to occur in females who had had two or more CS. Compared to patients with a similar number of previous cesarean sections, patients who sustained small bowel injuries in the index cesarean section were twice as likely to have had adhesiolysis of flimsy or dense lesions in the immediate preceding procedure.

Conclusion

Bowel injury during peritoneal entry at cesarean section is rare but may be frequently encountered in maternity units with high-volume CS rates.

## Introduction

Cesarean sections (CS) are among the most frequently performed obstetric surgical operations across the world [1]. The small bowel normally is outside the operative field during cesarean section, and encountering it just on peritoneal entry (despite routine steps for safe entry into the abdomen) is generally unexpected. In some patients, however, this unfortunately happened. Although rare, this complication may lead to wound complications, prolonged hospitalization, and medicolegal proceedings against the obstetrician or the hospital.

Routine steps for safe entry into the abdomen are one of the cornerstones of good surgical practice. The area of access is normally chosen to avoid damage or trauma to adjacent or underlying organs. It is known, however, that anatomical differences or abnormal fixation of an organ (frequently following prior surgery) increases the risk of inadvertent organ trauma during surgical procedures.

This risk is not commonly mentioned in the standard pre-cesarean patient consent [2], as it is deemed relatively infrequent.

One literature review [3] of Medical Literature Analysis and Retrieval System Online (MEDLINE)-cited publications related to post-cesarean complications cites the frequency of bowel injury ranging between 0.06% and 0.34%. It was pointed out that there was a relationship between the increasing number of bowel injuries and the increasing number of previous cesarean sections. In the paper, more focus was given to the incidence and “burden” of post-cesarean peritoneal adhesions. This was also a possible contributory factor to the likelihood of bowel injury, as adhesions may cause a fixation to a site in the lower abdomen where the obstetrician was not expecting to encounter the bowel.

This case-control retrospective study was designed to document the relative frequency and clinical features of patients who had small bowel injuries during peritoneal entry at cesarean section.

## Materials and methods

This was a case-control observational study. The study was based on patients who sustained inadvertent injuries to their small bowels during peritoneal entry at a repeat cesarean section. Control subjects were matched for the number of previous cesarean sections, who did not have small bowel injuries, and whose details were entered following the index patient.

Eligible patients include those who had their index cesarean sections at the old Women’s Hospital and the current Women’s Wellness and Research Center, Doha, Qatar, between January 1, 2015, and December 31, 2020.

Exclusion criteria were patients’ records lacking adequate information, patients with injuries at times not related to the time of peritoneal entry, and patients who had a delayed presentation with complications of small bowel injury during cesarean section, which was not identified ab initio.

The study was approved by our Institutional Research Review Board (MRC-01-21-212), after which we obtained a list of patients who reportedly sustained small bowel injuries during cesarean section from the Medical Records Department.

This study was conducted in accordance with the relevant hospital guidelines/regulations. Since this was a retrospective descriptive study and subjects had been de-identified before analysis, consent from patients was waived by the Institutional Research Board.

We went through the CERNER electronic records to ascertain the fulfillment of inclusion or exclusion criteria.

We extracted information about previous and index cesarean sections, whether the patient had had a non-cesarean laparotomy, and if the previous/proximate cesarean section was performed in-country or outside the country of our current practice. This was to enable us to assume the uniformity of cesarean section performance standards, presuming that this may not be necessarily so, elsewhere.

We also extracted information from the study group related to the ease of abdominal/peritoneal surgical access and if there were coexisting surgical problems before the index cesarean section. The score of the extent of small bowel injury was based on the American Association for the Surgery of Trauma (AAST) Small Bowel Injury Scale: Grade I, when there was hematoma (bruise or hematoma without injury) or laceration with partial thickness, without perforation; Grade II, characterized by a laceration of less than 50% of the circumference; Grade III, laceration of more than 50% of the circumference without transection; Grade IV, transection of the small bowel; and Grade V, transection of the small bowel with loss of segmental tissue or vascular injury with segmental devascularization.

Finally, postoperative hospital stay days were noted from the patient’s discharge summary document.

Statistical analysis was done using Statistical Package for the Social Sciences (SPSS) (IBM SPSS Statistics, Armonk, NY, USA). The Mann-Whitney U test and chi-square test were conducted to compare historical, operative parameters and the frequency of the clinical complication under consideration. Twelve instances of small bowel injury were recorded between 2015 and 2020 in the operating theater log books, of whom 10 fulfilled our eligibility criteria. The EpiCalc (online) tool (https://www.openepi.com/SampleSize/SSCC.htm) was used to calculate the sample size for this study. For a 95% confidence level, 80% power, and ratio of controls of cases of 3 to 1, with the 10 identified cases (study subjects), 30 controls were required.

## Results

Between January 1, 2015, and December 31, 2020, there were 99,367 deliveries in our facility, of which 31,677 were by cesarean section. We considered each episode of cesarean delivery as discrete, although some of the women had had up to five consecutive events. Of the 12 instances identified, a small bowel injury occurred during additional procedures to retrieve a translocated intrauterine contraceptive device in one patient; it was injured in the course of a difficult cesarean hysterectomy. Based on the 10 confirmed, a peritoneal entry small bowel injury rate of 0.0003 (three per 10,000 cesarean sections) was arrived at.

Table 1 is a summary of comparative demographic parameters between women who sustained small bowel injuries during peritoneal entry at cesarean section and those who did not. The mean age in years, the median number of previous caesareans, and the proportion of cesarean sections performed (locally) were comparable between the cases and controls. The median parity for cases was three (compared to the control median of two) (P value = 0.03). The odds of the cases being performed as an emergency procedure was 6.5 times when compared to the controls (50% versus 13%) (P value = 0.029).

**Table 1:** Comparison of selected demographic parameters between women who had bowel injuries and those who did not. CS: cesarean section, OR: odds ratio

Demographics	CS with bowel injury (N = 10)	Controls (N = 30)	P value
Age (mean, years)	33.9	32.5	0.4736
Parity (median)	3	2	0.030
Number of previous cesarean sections (median)	2	2	1.000
Emergency cesarean (number, %)	5 (50)	4 (13.3)	OR = 6.5, 0.029
Last cesarean section outside the country (number, %)	6 (66.67)	18 (66.67)	1.000

In Table 2, there was no difference noted in the median time after the previous cesarean, the availability of previous operative details, and the history of adhesions in the previous caesareans between the cases and the controls. Almost 60% of the controls had no complications in the previous surgeries when compared to only 30% of the cases (P value = 0.048).

**Table 2:** Key obstetric variables.

Obstetric variables	Cesarean section with bowel injury (N = 10)	Controls (N = 30)	P value
Time since previous cesarean section (median, years)	3.5	3	0.9122
Number of non-cesarean laparotomies (median)	0.5	0	0.006
Previous operative details available (number, %)	5 (55.56)	17 (62.96)	0.712
No complications in previous surgery (number, %)	3 (30)	17 (56.67)	0.048
Previous adhesions (number, %)	0 (0)	7 (23.33)	0.274

Procedure-related details in patients who had small bowel injuries are summarized in Table 3.

**Table 3:** Procedure-related details in patients who had small bowel injuries during peritoneal entry at cesarean section. CS: cesarean section, AAST: American Association for the Surgery of Trauma

Entity	N = 10	Comments
Mean age (years)	33.9 years	
Number of previous CS		
None	1	The patient had had a myomectomy.
1	2	One of the patients had had a re-laparotomy after a prior cesarean section.
2	4	
3	2	
Previous CS was performed locally	6	
Documented findings on peritoneal entry		
Small bowel “matted” to the anterior abdominal wall	7	
Small bowel and uterus densely adherent to the anterior abdominal wall	2	
Dense anterior abdominal wall adhesions	1	
AAST grade of small bowel injury		
Grade I: laceration with partial thickness without perforation	7	
Grade II: laceration of less than 50% of the circumference	2	
Grade III: laceration of more than 50% of the circumference without transection	1	
Mean duration of hospital stay after index CS (days)	4.6 days	

All injuries involved the ileum. The duration of hospital stay post-cesarean was not related to any surgical events thereafter.

## Discussion

The reasons why the small bowel adheres to the post-cesarean parietal peritoneum in some patients are unclear.

The low frequency of bowel involvement in post-cesarean adhesive disease is often attributable to the physical barrier of a recently gravid uterus, between the bowel and the anterior lower transverse uterine incision, and the bladder falls back over the uterine incision.

Whether the closure of the parietal peritoneum or not, the nature of the suture used for re-peritonealization, and whether the parietal peritoneum was entered sharply or bluntly have all been explored with relation to general and gastrointestinal surgery and to the performance of cesarean sections [3-7]. One possible etiology of direct or serosal abrasion trauma to the small bowel could be the manipulation and retraction by surgical sponges or gauze or surgical towels [8].

Although the risk of adhesive bowel and bladder disease increases with the number of repeat cesarean sections [9-11], our small series suggests that lower-order surgeries are not exempt. In the study from Saudi Arabia [11], only one instance of bowel injury (in a cluster sample of 318 higher-order cesarean sections) was reported, compared to six bladder injuries.

In a summative data analysis from the USA [12], bowel injury occurred in the course of seven out of 6,201 primary cesarean sections.

When inadvertent bowel injury occurs during peritoneal entry at cesarean section, the essential step is to isolate the segment of the traumatized bowel and ensure containment of the spill [13]. The baby should be delivered, and the assistance of a general or colorectal surgeon should be sought.

In published articles, frequently by implication, there is a tendency to attribute bowel injury during cesarean section to an obstetrician not being “careful” or “meticulous” enough. The medicolegal implications of using such terms may not be lost on the patient, her relations, or a “negligence claims lawyer.”

One online medicolegal opinion went thus, “A bowel injury at the time of cesarean is not in itself evidence of malpractice or performance below the standard of care. Bowel injuries have been reported and can occur even in the best of hands” [14].

Good clinical practice requires that patients who are particularly at risk be preoperatively made aware of this possibility [15].

Obstetricians performing cesarean sections through a previously scarred lower abdominal scar will assume the presence of a high-riding (adherent) bladder and therefore aim to enter the peritoneal cavity as “high” as safely possible. Such an avoidance maneuver is unlikely to be of value in the presence of an unexpected presence of an adherent loop of the small bowel. The majority of the numbers of small bowel injuries in this series were Grade 1 AAST, which attests to due reasonable care at peritoneal care despite the occasional inevitability of trauma.

It is noteworthy that, even while using optically facilitated trocar peritoneal entries during laparoscopy, there were 18 bowel perforations among the 79 major complications cited over nearly 20 years of use, second only to vascular injuries [16].

In a longitudinal study of 25,354 women who underwent a hysterectomy in Sweden over a period of 14 years [17], previous cesarean section was a predictor of bowel injury, with adjusted odds of 1.87, while other abdominal surgery had adjusted odds of 2.27 for bowel injury.

It would be desirable to detect the presence of a bowel that is adherent to the anterior abdominal wall before a repeat cesarean section. A group from Munich, Germany [18], used a nine-segment abdominal mapping with visceral slide on a 1.5 Tesla system MRI in 90 patients with previous abdominal surgery. The most common adhesions were between the anterior abdominal wall and small bowel loops (32.5%). Comparing the MRI diagnosis and intraoperative findings, there was an overall MRI accuracy of 89%, with wide ranges in both sensitivity and specificity for comparisons between different segments.

Since practically most obstetric patients in modern practice would have an ultrasound scan sometime in the third trimester of pregnancy, screening for a suggestive “sliding sign” associated with the possible presence of intra-abdominal adhesions [19] is an attractive proactive strategy that may indirectly reduce the risk of bowel injury during cesarean section.

One surgical classification of intra-abdominal adhesions after a previous laparotomy [20] describes “organs strongly attached to severe adhesions, (with) damage of organs hardly preventable.” Inadvertent small bowel injury during peritoneal cesarean section may well be in this category.

Inadvertent small bowel injury during peritoneal entry at cesarean section is uncommon, even in a high surgical-risk obstetric population. It may therefore be difficult to undertake a study that will prove or justify the routine use of adhesion-preventing measures [21,22] for this specific entity.

The design and conduct of this retrospective medical records review meet the criteria for methodological quality for case series. Ascertainment was defined by the selection and ascertainment criteria. Within the defined domains, we were able to attribute the plausibility of causality, with no alternative explanations for the outcomes. The details would appear sufficient for the surgeon or obstetrician to infer similar findings from their practice records.

## Conclusions

Inadvertent small bowel injury during peritoneal entry at cesarean section is uncommon and largely unpredictable, even in a high surgical-risk obstetric population. Obstetricians must be aware of this, and that recognition should be followed by appropriate management and patient communication.
